# Diarrhea of Children

**Published:** 1893-10

**Authors:** 


DIARRHEA OF CHILDREN.—In treating diarrhea of infants, children or adults we should always remember that the secretions are defective, as indicated by a dry or coated tongue, unnatural color of stools, etc., and to attempt to arrest the watery discharges with such a pathological condition present by opiates and astringents will not last—will do injury, harm instead of good. Therefore first use:

℞. Calomel.................gr. j.
Sodii bicarb.............gr. v.
Pulv. sacch. alb..........gr. xx.

M. ft. chari. No. x. Sig. One every two or three hours until discharges are changed in color and consistency; or hydrarg. cum cretæ, one part, triturated with two or three parts of sacch. lacti. Of this powder give two grains every two or three hours. These powders will often restore healthy secretory action, and cure the diarrhea alone. If not, follow with small doses of bismuth, sub. carb., nux vomica and ipecac, or a few drops of the following:

℞. McMunn's elixir oppii...
Tinct. rhei.............
Tinct. camphoræ, aa.... ℥ ss.

M. Sig. From five to ten drops every hour or two, as needed, and according to age of child; or for very young children prescribe:

℞. Syr. rhei. aromat....... ℥ j.

Tinct. opii campn...... ℥ ss.

Tinct. cardamon comp.. ʒ ij.

Aquæ calcis............ ℥ vj.

M. Sig. Teaspoonful every hour or two, as needed.—*Livezey, Ex.*

